# The correlates of after-school sedentary behavior among children aged 5–18 years: a systematic review

**DOI:** 10.1186/s12889-015-2659-4

**Published:** 2016-01-22

**Authors:** Lauren Arundell, Elly Fletcher, Jo Salmon, Jenny Veitch, Trina Hinkley

**Affiliations:** Centre for Physical Activity and Nutrition Research, Deakin University, 221 Burwood Highway, Burwood, Australia

## Abstract

**Background:**

Children and adolescents spend a large proportion of the after-school period in sedentary behaviors (SB). Identifying context-specific correlates is important for informing strategies to reduce these behaviors. This paper systematically reviews the correlates of children’s and adolescents’ after-school SB.

**Methods:**

A computerized literature search was performed in October 2015 for peer-reviewed original research journal articles published in English before October 2015. Eligibility criteria included: 1) sample aged 5–18 years; 2) quantified the amount of SB or component of this that the children/adolescents were performing after school; 3) a measure of SB as the dependent outcome; and 4) the association between potential correlates and after-school SB.

**Results:**

Data were synthesized in October 2015. Thirty-one studies met the eligibility criteria: 22 studies among children (≤12 years), six among adolescents (>12 years), two had a combined sample of children and adolescents and one cohort followed children from childhood to adolescence. Findings were separated by after-school location i.e. after-school programs (*n* = 4 studies) and unidentified locations (*n* = 27). There was insufficient evidence to draw conclusions on all but two of the 58 potential correlates: sex and age. Among children at unidentified locations there was a null association between sex (male) and overall after-school SB, a null association between sex (male) and after-school TV viewing, a positive association between age and overall after-school SB and an inconsistent association between age and after-school TV viewing. No correlates of after-school sedentary behaviour while at after-school programs were identified.

**Conclusions:**

Only two correlates have been investigated frequently enough to determine an overall association; neither correlate is modifiable. Due to the lack of consistent investigation of potential correlates, further evidence is required to accurately identify potential intervention targets.

**PROSPERO registration number:**

CRD42014009180

**Electronic supplementary material:**

The online version of this article (doi:10.1186/s12889-015-2659-4) contains supplementary material, which is available to authorized users.

## Background

Sedentary behaviors are defined as behaviors expending ≤1.5 metabolic equivalents (METS) in a sitting or reclining posture (e.g. TV viewing, computer use, reading) [1]. The total time spent engaged in sedentary behaviors is called sedentary time. Emerging evidence shows the health and other risks of engaging in elevated amounts of sedentary behavior among youth, such as increased adiposity, decreased fitness, poor self-esteem and poor academic achievement [2–4]; however, this evidence is at times equivocal [5]. Despite this, approximately 75 % of youth in many developed countries exceed Government recommended levels of no more than two hours of recreational screen time per day [6–8].

The after-school period, from the conclusion of school until 6 pm [9], is typically characterized by children engaging in sedentary behaviors. Up to 38 % of this period is spent sedentary [10] and children watch over 70 % of their daily TV between 3–9 pm [11]. Interventions targeting after-school sedentary behaviors may therefore be effective. Children are not restricted by the school timetable during this period, and may have some discretionary choices between active or sedentary options. Although warranted, such interventions require an understanding of context-specific correlates of participation in these behaviors during the after-school period prior to development.

Previous reviews exploring the correlates of children’s individual sedentary behaviors such as total screen-viewing [12] and TV viewing [13], and overall sedentary time (objectively and subjectively measured) [14], have examined children’s daily behaviors without specific attention to the after-school period. As there are health outcomes specific to screen-viewing [3, 4, 15], and these behaviors are often intervention targets [16, 17], investigation into the correlates of after-school screen-viewing behaviors as well as total sedentary time during this period is important. In addition, the context of the after-school period (i.e. location of the child and who the child is with) is likely to be different to what children experience during the whole school day. Therefore, it is likely that the correlates of sedentary behaviors performed after school vary from the correlates of daily sedentary behaviors.

Many theories have been used to facilitate the study of behaviors and their corresponding correlates. Ecological models posit that behavior is influenced by intrapersonal/demographic factors as well as their social/cultural and physical/policy environments [18], all of which are likely to impact a child’s after-school sedentary behavior. The aim of this paper is to systematically review the correlates of children’s and adolescents’ after-school sedentary behavior organised according to an ecological framework.

## Methods

This review is registered with PROSPERO (registration number: CRD42014009180).

### Search procedure

Using the EBSCOhost search engine, a computerized search for literature was performed in October 2015 within the following databases: Academic Search Complete, CINAHL Complete, Education Research Complete, MEDLINE, MEDLINE Complete, PsycARTICLES, Psychology and Behavioral Sciences Collection, PsycINFO and SPORTDiscus with Full Text. Peer-reviewed original research journal articles published in English until October 2015 were sought. The following keyword combinations were used for *age* (school age, youth, young, child*, adolescen*), *behavior* (sedentar*, television, TV, screen, electronic games, inactiv*) and *time-of-day* (after-school, after school, afternoon, evening, critical window, critical hours).

Articles were analysed in October 2015 and were included if they met the following criteria: 1) included children aged 5–18 years; 2) quantified the amount of sedentary time/behavior the children were performing after school (in minutes or proportion of the period); 3) included a measure of sedentary behavior (objectively or subjectively measured) as the dependent outcome; and 4) assessed the association between potential correlates and after-school sedentary behavior. Studies that included special populations (e.g. children with a disability or overweight/obese populations) were excluded due to the inability to generalise the findings to the broader population. No restriction was placed on the definition of ‘after-school’ to allow for the greatest inclusion of studies. Articles that examined sedentary behaviors ‘outside of school’ were excluded as that definition typically includes behaviors before school and on weekends which was beyond the scope of the current review. All sedentary behaviors were reported (e.g. TV viewing, Computer/ DVD/video game use) to enable an exploration of the potential correlates specific to each behavior. Articles were included regardless of where the children were located during the after-school period (e.g. at after-school program, at home) to allow comparisons of behaviors and potential correlates.

Initially, each title and abstract was reviewed to determine eligibility. The full-text of studies deemed eligible were retrieved and assessed. Relevant papers from other sources (e.g. reference lists) were also added if eligible. All articles were reviewed by two authors (LA and EF) and any differences were discussed until agreement was achieved (83 % agreement in initial screening). When eligibility was unclear contact was attempted with the corresponding author for further clarification (*n* = 4 authors).

### Methodological quality and risk of bias assessment

Study quality and risk of bias was determined using a modified published rating scale from McMaster University [19]. Six methodological components were assessed including selection bias (e.g. sample representativeness), study design (e.g., RCT), confounders (e.g., were between-group differences controlled for?), blinding (e.g. was the outcome assessor aware of group allocation), data collection methods (e.g. are they valid and reliable), and withdrawals and dropouts (e.g. percent of participants completing/providing full data). Intervention-specific criteria within any component was not assessed for observational studies (e.g. intervention integrity, blinding). As recommended in the quality assessment tool’s dictionary, each study was given a score of weak, moderate or strong for each component. Two reviewers (LA and EF) independently assessed the quality of each study, compared results and discussed any differences until agreement was achieved (93 % agreement in initial study quality assessment). The PRISMA guidelines were followed [20].

## Results

**Fig. 1:**
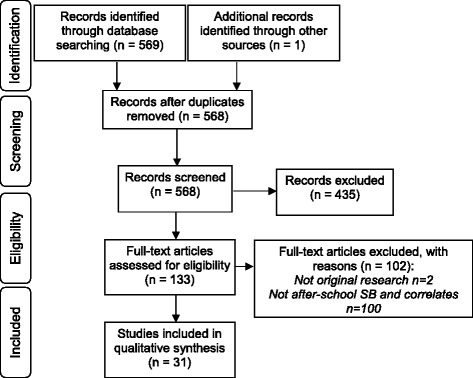
Flow chart of search results

Figure 1 shows that 569 articles were identified, screened and assessed for eligibility. Of these, 31 met the inclusion criteria. Studies were analysed for children and adolescents separately. Studies were included in the child data when the mean age was ≤12 years [10, 11, 21–39] (*n* = 22) and in the adolescents’ data when the mean age was >12 years [40–45] (*n* = 6). One cohort studied examined correlates when the sample was children (9–10 and 10–11 years) and adolescents (13–14 years) [46]. For this study, data for the 9–10 and 10–11 year olds were included in the children’s findings and data for the 13–14 year old were included in the adolescents’ findings. Two additional studies had a sample that included both children and adolescents (grades 3 and 9 [47] and 9–15 years [48]). These two studies combined the age groups, therefore their findings were duplicated to be included in both the child and adolescent data for this review as the potential correlates related to both age groups. Therefore, a total of 25 studies were included for children and nine for adolescents. Study characteristics can be found in (Additional file 1: Table S1).

### After-school period definitions

A variety of definitions for the ‘after-school’ period was provided and many studies did not define the period. Among the four studies examining potential correlates of sedentary behavior while children were at after-school programs, only one study [21] provided information to define ‘after-school’. However, that study defined the period as the average number of minutes (208, range 60–240) after school and did not provide a start and/or finish time, and it is assumed that this would have varied widely by child. The other three studies did not report the actual time period [26, 29, 31]. Among the 27 studies examining correlates of children’s/adolescents’ after-school behaviors while at unidentified locations, four did not provide a definition [33, 45, 47, 48]. Twenty different definitions of the after-school period were used among the remaining 23 studies ranging from two hours immediately after school [36, 37] through to 12noon–9 pm [32].

### Child’s location

Only four studies that met the inclusion criteria assessed the potential correlates of children’s sedentary behavior while attending an after-school program. All four studies were among children [21, 26, 29, 31]. Of the remaining studies, 22 did not report where the children/adolescents were located after school [10, 11, 22–25, 28, 32, 33, 36–41, 43–49] and five reported that the children/adolescents were at a variety of locations after school [27, 30, 34, 35, 42]. For synthesis of the results, these 27 studies were combined to represent children/adolescents at ‘unidentified locations’.

### Country of study

All four studies investigating the potential correlates of children’s/adolescents’ sedentary behavior during after-school programs were conducted in the United States [21, 26, 29, 31]. The majority of the studies investigating after-school behaviors while at unidentified locations were conducted in the United States (*n* = 12) [11, 22, 23, 27, 30, 34, 35, 41–43, 45, 48]. The only other countries from which multiple studies were identified were the United Kingdom [28, 32, 39, 40, 46, 49], Australia [10, 24, 33] and Canada [36, 37]. One study was identified from each of the following: Portugal [44]; a combined sample from Bulgaria, Taiwan and the United States [25]; a combined sample from England and Spain [38]; and a combined sample from Denmark, Portugal, Estonia and Norway [47].

### Measurement tools

Data collection methods for the studies examining the potential correlates of children’s/adolescents’ sedentary behavior while at after-school programs were predominantly objectively measured by an ActiGraph GT1M accelerometer with a sedentary behavior cut point of <100 counts per minute (cpm) [21, 29, 46, 49] or <1.5METS [31]. One study used self-report physical activity/sitting activities recall [26].

Among the 27 studies examining after-school sedentary behavior while at unidentified locations, 16 used objective measures including Actical accelerometer (<1.5METS [23]), ActiGraph accelerometer with several sedentary cutpoints (<50 cpm [44], <100 cpm [10, 24, 28, 30, 32, 38, 43, 46, 49], <288 cpm [39], <300 cpm [36, 37], and <800 cpm [41]), and direct observation (Children’s activity rating Scale [CARS]) [22]. Eleven studies used subjective measures, only one of which used a parent proxy-report child sedentary behavior log (watching TV and computer/video games including handheld devices in 15-min intervals) [11]. The remaining 10 studies used child self-report: behaviors at 15 min intervals [40], TV viewing (60 min blocks) [47], self-report survey of behaviors in 60 min intervals [25], screen time recall (30 min blocks) [33], increasing intervals of a variety of sedentary behaviors (i.e. 15, 30 then 60 min) [45], a sedentary behavior diary (every 20 min) [42], the Self-Administered Physical Activity Checklist (SAPAC) [48], and behavior recall via telephone interview (15 min intervals) [27, 34, 35]. Studies were classified as measuring overall sedentary behavior, TV viewing, computer/DVD/video games, a composite measure of screen-based sedentary behaviors (when TV viewing and computer/DVD/video games may have been included in the measure but were unable to be extracted for separate analysis) or non-screen-based sedentary behaviors.

### Determining an association

A previously published coding model [50, 51] was used to determine the overall association between a correlate and outcome behavior (‘+’ positive, ‘-’ negative or ‘0’ no/non-significant association). When there were four or more findings that investigated a correlate and sedentary behavior or a component of sedentary behavior, the association was assigned ‘0’ (null: 0–33 % of findings supported an association), ‘?’ (inconsistent: 34–59 % of findings supported an association) or ‘+’ or ‘–’ (positive or negative: 60–100 % of findings supported an association).

### Correlates of children’s and adolescents’ after-school sedentary behavior

#### Correlates of children’s after-school sedentary behavior

In total, 58 potential correlates of children’s after-school sedentary behavior were identified from the literature [10, 11, 21–39, 42, 47, 48]. Of these, only sex and age were assessed frequently enough to provide an overall association. Table 1 shows a null association between sex and overall sedentary behaviors after school [10, 11, 24, 31, 32, 35, 39]. There was also a null association between sex (being male) and after-school TV viewing [25, 27, 47]. Age was positively associated with overall sedentary behaviors [10, 35, 46, 48, 49], and inconsistently associated with after-school TV viewing [27, 47]. No overall associations were found with any potential correlates of children’s sedentary behavior while at after-school programs.

**Table 1:** Correlates of children’s after-school sedentary behavior reported in ≥4 findings

	Overall sedentary behavior		TV viewing	
Correlate variables	Ass.	Studies	Ass.	Studies
Sex (male)	+		+	[27]^a^
	-	[32]	-	
	0	[10, 11, 24, 35, 39] [31]^h^	0	[25], [27]^b^, [47]
Overall association (≥4 findings)	0		0	
Age	_+_	[10, 35, 46, 48, 49]	+	[27]^a^, [47]^cd^
			0	[27]^b^, [47]^efg^
Overall association (≥4 findings)	_+_		?	

Children aged 5–12 years

*Abbreviations*: *‘0’* null association, *‘+’* positive association, *‘-’* negative association

^a^African American sample

^b^White sample

^c^All sample, includes children from Denmark, Portugal, Estonia and Norway

^d^Estonia sample

^e^Denmark sample

^f^Norway sample

^g^Portugal sample

^h^Sample at after-school programs

Table 2 shows the potential correlates of children’s after-school sedentary behavior that were measured too infrequently to determine an association. Overall sedentary behavior was the mostly commonly assessed outcome with 36 potential correlates assessed for their association. This was followed by TV viewing (15 potential correlates), non-screen-based sedentary behaviors (8 potential correlates), screen-based sedentary behaviors (6 potential correlates and computer/DVD/video games (4 potential correlates). Five potential correlates (age, sex, BMI, race and family structure) were assessed across multiple behavior outcomes.

**Table 2:** Correlates of children’s sedentary behavior that were reported in <4 findings (insufficient evidence)

Overall sedentary behavior	TV viewing	Computer/DVD/video games	Screen based SB	Non-screen based SB
Intrapersonal	Intrapersonal	Intrapersonal	Intrapersonal	Intrapersonal
At ‘unidentified locations’	At ‘unidentified locations’	At ‘unidentified locations’	At ‘unidentified locations’	At ‘unidentified locations’
+[48] Race (non-Caucasian)	+[47]^ceg^ /0[22]^,^ [47]^df^ BMI	+[25, 27]^b^/0[27]^a^Sex (male)	+[33–35] Sex (male)	-[27]^,^ [34, 35] Sex (male)
+[11]^h^/0[11]^i^ AS MVPA	+[48]Race (non-Caucasian)	+^34^/0[27] Age	+^34^/0^33^ Age	+[34, 35]/0[27] Age
+[28] Parental education	+[47]^cef^/0[47]^gd^ Child behavior autonomy	Social	+[34] Race (non-Caucasian)	0[34] Race (non-Caucasian)
+[32] Sometimes eats breakfast (ref always eat)	-[47]^ce^/0[47]^dfg^ Father’s income high (ref low)	0[27] Family structure (1 parent house)	Social	Social
-[37] Child IM: high (ref low)	0[22] Sum of skinfolds	Physical/Policy	At ‘unidentified locations’	At ‘unidentified locations’
-[28] Deprivation (ref least deprived)	0[22] Waist:Hip ratio	−0[25] Country (Bulgaria, Taiwan, USA)	-[34] With peer group AS	+[34] With mum/dad AS
0[28] No. of cars at home	0[47] Father’s income med (ref low)	0[27]Attends AS Program (ref other care)	Physical/Policy	+[34] With unrelated adult AS
0[28] Household income	0[47] Mother’s income high/ med (ref low)		At ‘unidentified locations’	0[27] Family structure (1 parent house)
0[32] Poor/Good quality breakfast eaten (ref none eaten)	Social		-[34] Time in public place AS	Physical/Policy
At after-school program	0[27] Family structure (1 parent house)		-[34] Time outside at home AS	At ‘unidentified locations’
-[29] AS MVPA				-[34]Time outside at other house (not own)
0[26, 31]^j^ BMI	Physical/Policy			+[27]Attends AS Program (ref other care)
Social				
At ‘unidentified locations’	At ‘unidentified locations’			
0[23] Mother’s SB	+[47] Country: Portugal, Estonia, Norway (each ref Denmark)			
0[23] Father’s SB	0[25] Country (Bulgaria, Taiwan, USA)			
Physical/Policy	+[47] TV environment			
At ‘unidentified locations’	+[47]^d^/0[47]^cefg^ No. TV sets in the home			
-[28] Home has private garden	0[47] TV in bedroom			
-[36]^h^/0[36]^i^ Outdoor play	-[27]Attends AS Program (ref other care)			
-[30]^i^/0[30]^h^ Attends AS Program (ref home)				
-[44]^h^/0[44]^i^ Season (winter)				
0[38] Country:England (ref Spain)				
+[46] Rainfall				
At after-school program				
+[21]^h^/0[21]^i^ AS Program has PA policy				
+[21] AS Program collects feedback on activities children want				
+[21]^h^ AS Program staff provided with 1-4 h of PA training				
+[21]^h^/0[21]^i^ AS Program undergone ≥1 year of evaluation				
+[29] Duration of AS Program session				
+[29] AS Program conducted inside				
-[21]^i^/0[21]^h^ AS Program provides activities for both sexes				
-[29] ≥25 % of AS Program allocated to PA				
0[21] AS Program provides age and skill appropriate activities				
0[29] AS Program provides organised/free play activities				
0[29]AS Program provides non-fixed equipment				
0[29] No. of children				
0[29] AS Program boys:girls ratio				
0[29] AS Program staff:children ratio				
0[21] Presence of parent workshop promoting importance of PA				
0[21] AS Program curriculum undergone formal evaluation				

*Abbreviations*: *‘0’* null association, *‘+’* positive association, *‘-’* negative association, *AS* after school, *MVPA* moderate- to vigorous-intensity physical activity, *SB* sedentary behavior, *IM* independent mobility

^a^African American sample

^b^Caucasian sample

^c^All sample, includes children from Denmark, Portugal, Estonia and Norway

^d^Estonia sample

^e^Denmark sample

^f^Norway sample

^g^Portugal sample

^h^Male

^i^Female

^j^sample at after-school programs

Of the potential correlates assessed for their association with overall sedentary behavior, 12 were intrapersonal (e.g. age, race), two related to the social environment (e.g. mum’s/dad’s sedentary behavior) and 22 related to the physical/policy environment (e.g. number of TV sets at home). However, 16 of these physical/policy environment correlates were assessed among children attending after-school programs and are specific to that program. Among the correlates of after-school TV viewing, eight were intrapersonal, one was social and six were related to the physical environment. Computer/DVD/video games had five potential correlates, two intrapersonal, one social and two from the physical environment. The social and physical environment correlates of children’s after-school screen-based and non-screen-based sedentary behaviors examined who they were with (i.e., mum, dad, unrelated adult) and where they were located (i.e., public place, outside at home, outside at other home).

#### Correlates of adolescents’ after-school sedentary behavior

No correlates of adolescents’ after-school sedentary behavior were measured often enough to determine an overall association. The 15 potential correlates of adolescents’ after-school sedentary behavior are shown in Table 3. Among adolescents, TV viewing was the behavioral outcome most frequently assessed with 11 potential correlates assessed for their association. Seven potential correlates were assessed for their association with overall sedentary behavior, two were assessed with computer/DVD/video games, and one with non-screen based sedentary behavior. Only sex, race and BMI were assessed across multiple behavior outcomes. Among all behaviors, the majority of the potential correlates were intrapersonal (*n* = 9) and TV viewing was the only behavioral outcome with which physical/policy environment correlates were assessed.

**Table 3:** Correlates of adolescents’ sedentary behavior that were reported in <4 findings (insufficient evidence)

Overall sedentary behavior	TV viewing	Computer/DVD/video games	Non-screen based SB
Intrapersonal	Intrapersonal	Intrapersonal	Intrapersonal
-[41]Sex (male)	0[40] Sex (male)	+[40] Sex (male)	0[40] Sex (male)
+[48] Age	+[47]^ace^/0[47]^bd^ BMI	+[45]^(comp/int)^/-[48]/0[45]^(vid games)^ Race (non-Caucasian)	
+[43]/-[42]BMI	+[48]/0[45] Race (non-Caucasian)		
+[48] Race (non-Caucasian)	+[47]^acd^/0[47]^be^ Child behavior autonomy		
+[43] Body Fat %	-[47]^ac^ /0[47]^bde^ Father’s income high (ref low)		
Social	0[47] Father’s income med (ref low)		
+[42] Time supervised AS	0[47] Mother’s income high/med (ref low)		
+[42] Time alone AS	Physical/Policy		
Physical/Policy	+[47] TV environment		
0[46] Rainfall	+[47]^b^/0[47]^acde^No. TV sets in the home		
	0[47] TV in bedroom\		
	+[47] Country: Portugal, Estonia, Norway (each ref Denmark)		

*Abbreviations*: *‘0’* null association, *‘+’* positive association, *‘-’* negative association, *AS* after-school, *BMI* Body Mass Index

^a^All sample, includes children from Denmark, Portugal, Estonia and Norway

^b^Estonia sample

^c^Denmark sample

^d^Norway sample

^e^Portugal sample

### Methodological quality and risk of bias assessment

The majority (67 %) of the studies were cross-sectional [11, 23–26, 28–33, 38, 40–44, 47, 48] with 11 cohort studies identified [10, 21, 22, 27, 34–37, 45, 49] and one study used baseline data from three interventions targeting overall physical activity [39]. Reliability and validity of measurement tools was poorly reported with only eight studies reportedly using valid and reliable tools [22–24, 26, 30, 31, 42, 44]. Only six studies had a low selection bias with a “very/somewhat likely” representative sample and ≥80 % selected individuals agreeing to participate [24, 27, 31, 33, 41, 43]. Completion rates were generally high with over 80 % completion in ten studies [24, 25, 31, 32, 36, 37, 41–43, 48] and 60–79 % completion in a further eleven studies [10, 23, 27, 30, 33–35, 38, 44, 45].

## Discussion

Fifty-eight potential correlates of children’s and adolescents’ after-school sedentary behavior were identified in this systematic review. As found in previous reviews of daily sedentary behavior among children and adolescents [52, 53], there was insufficient evidence to draw conclusions about the majority of these. Only two variables (sex and age) were assessed frequently enough (four or more times) to produce an overall association. Both of these variables were non-modifiable, were identified from studies among children, and were within the intrapersonal domain of the ecological model.

The complex nature of after-school sedentary behaviors is highlighted within these findings due to the null associations between sex (male) and overall sedentary behavior and a null association with TV viewing. These findings are in contrast to a previous review that found an inconsistent association between sex and overall sedentary behavior among preschool-aged children [14], but concur with reviews among preschool children [14] and 2–18 year olds [13] that found no association between sex and daily TV viewing. Another review of the correlates of adolescents’ (13–18 years) daily sedentary behaviors found a positive association with sex (male) [52]. However, the authors grouped their studies so that the association was reported for combined TV/video/computer use or TV viewing without being able to determine if the association existed for each specific sedentary behavior. This behavior grouping may have been to align with public health recommendations, so future research may benefit from identifying discretionary sedentary behaviors (e.g. computer use for recreation) over non-discretionary sedentary behaviors (e.g. computer use for homework) for targeted interventions.

This review also found a positive association between age and children’s after-school overall sedentary behavior, an association previously seen with young children’s (≤7 years) daily screen-viewing [12]. In contrast to this was the inconsistent association between age and children’s after-school TV viewing. This also contradicts a previous cohort studies that found TV viewing declines with age while computer use increases [54]. The positive association with age and overall sedentary behaviour but inconsistency between age and TV viewing may be due to increase homework requiring computer use or increased participation in organised activities after-school. However, the mixed findings from this review highlight the need for further research to better understand how the use of individual components of sedentary behavior (i.e. TV viewing, electronic game use) varies as children age.

Despite evidence that sedentary time increases with age [55], a smaller number of potential correlates of after-school sedentary behavior were identified among adolescents than children in all three domains of the ecological model. This is a reflection of the lower number of studies among adolescents. Further it highlights the need for further investigation into the correlates of other components of sedentary behaviors such as homework/academics and recent technologies such as iPads and Kindles, particularly as the amount of suggested homework time increases as students progress through school [56].

The majority of the potential correlates of children’s overall sedentary behavior and the components of sedentary behavior while at unidentified locations were intrapersonal. Conversely, all of the potential correlates of children’s after-school sedentary behavior while at after-school programs were within the physical/policy domain of the ecological model. This suggests that correlates of sedentary behaviors performed during the after-school period may be dependent on the setting, the policies in place and features of the physical environment. Future research should identify the setting and context of children’s sedentary behavior in the after-school period.

### Limitations

There are a number of limitations within the literature included in this review that need to be acknowledged. Firstly, the majority of studies did not report where the child was located after school and these studies may have included children attending after-school programs. The correlates of children’s and adolescents’ after-school sedentary behavior may be specific to particular contexts or locations and therefore identifying differences between contexts is important for the development of intervention strategies. Secondly, international comparisons are difficult as the majority of the studies that met the eligibility criteria were among samples from the United States and the United Kingdom. Countries and cultures have different environments which may influence children’s and adolescents’ after-school sedentary behavior and so need to be further explored. Thirdly, consideration must be taken of the definitions of the after-school period used when examining the correlates of after-school sedentary behavior. The potential correlates of a child’s/adolescents’ behavior at 2 pm may vary considerably to those influencing the behavior at 8 pm. However, such differences were not able to be determined from the literature as all time frames were considered ‘after-school’. Fourthly, the variety of measurement tools and data management used may have impacted on the findings. For example, among the objective measures, a variety of sedentary cut points were used. This may influence the reported time children/adolescents spent in sedentary behavior [57] and subsequently may impact the correlates identified with this behavior. Further, objective techniques do not provide behavior-specific information, while subjective measures may be at risk of recall bias, particularly among children [58]. However, despite the limitations associated with each measurement tool, it is important to use both objective and subjective measures to identify associations between overall sedentary behaviors with individual health components. This information is vital given the emerging evidence of health outcomes associated with individual sedentary behaviors [3, 4, 15]. Finally, due to the diversity of samples, measures and variables used, we are unable to perform a meta-analysis.

Future research should focus on increasing the literature assessing correlates of children’s and adolescents’ after-school sedentary behavior from all domains of the ecological model using valid and reliable measurement tools, consistent terminology and a standardized after-school period definition. Additional attention on the correlates for adolescents would assist with intervention development among this understudied age group. Longitudinal studies among children and adolescents would also provide valuable information about the determinants of after school sedentary behaviors and sedentary time. Future studies comparing associations by sub-groups, such as sex, location and ethnicity would further assist tailoring intervention strategies targeting after-school sedentary behaviors and sedentary time.

## Conclusions

This review highlights the need for further research examining the intrapersonal, social/cultural and physical environmental/policy correlates of children’s and adolescents’ after-school sedentary behavior. There was insufficient evidence to draw conclusions about the majority of potential correlates with overall associations only observed for two non-modifiable variables among children: sex (male) and age. This lack of evidence makes the identification of potential strategies to decrease children’s and adolescents’ after-school sedentary behaviors challenging. Further investigation and identification of country-, context- and behavior-specific correlates of sedentary behaviors in both children and adolescents is required to develop effective interventions that target healthy levels of after-school sedentary behavior in these populations.

## Additional file

Additional file 1: Table S1. Study characteristics. (DOCX 19 kb)
