# Supplementary material for: The correlates of after-school sedentary behavior among children aged 5–18 years: a systematic review
Source: BMC Public Health. 2016 Jan 22;16:58. doi: 10.1186/s12889-015-2659-4 (PMC4722784; doi:10.1186/s12889-015-2659-4)
Supplement: Additional file 1: Table S1. — Study characteristics. (DOCX 19 kb) [file 12889_2015_2659_MOESM1_ESM.docx]

**Additional Table 1: Study characteristics**

| **Author** | **Year** | **Sample size** | **Age in years (range or M (SD)), gender (%male)** | **Country** | **After-school period definition** | **Study design** | **Location of children** | **SB measure** | **Quality score/ risk of bias** |
| --- | --- | --- | --- | --- | --- | --- | --- | --- | --- |
| Arundell et al.^8^ | 2013 | n=2053 | Group 1: n= 608, 5-6 years, 52% male Group 2: n= 1445, 10-12 years, 45% male | Australia | End of school bell-6pm | Cohort | Not reported | Actigraph 7164, 60 sec epoch, SED<100cpm | Mod Quality / Mod Bias |
| Atkin et al.^39^ | 2008 | n=1,484 | 13-16 years, 38% male | UK | 3.30-6.30pm | Cross-sectional | Not reported | Self-report diary of "free time" reporting behaviours, location and social context | Low Quality / High Bias |
| Bailey et al.^38^ | 2012 | n=135 | 11.7 (±1.4) (42% boys) | UK | End of school bell - 6.30pm | Baseline data from PA (intervention) | Not reported | RT3 triaxial accelerometers, 60 sec epoch, SED: <288 cpm | Low Quality / High Bias |
| Beets et al.^49^ | 2013 | n=785 | Kinder to year 5, 50% male | USA | Average of 208 minutes/day (range 60-240) | Observational | After-school care | Actigraph GT1M, 5sec epoch, SED <100cpm | Low Quality / High Bias |
| DuRant et al.^21^ | 1996 | n=138 | 5-6 years %male not reported | USA | >150mins after 3pm​ | Cross-sectional data from longitudinal study | Not reported | Observational : Children's activity rating Scale (CARS) | Mod Quality / Mod Bias |
| Fuemmeler et al.^22^ | 2011 | n=45 parent-child triads (mothers, fathers and children) | Child age: 10.6 (±0.7) 51% male. Mother age 40.6 (±5.6), father age: 42.8 (±6.2) | USA | 3-7pm | Cross-sectional | Not reported | Actigraph 7164, 60sec epoch, SED<1.5METs | Mod Quality / Mod Bias |
| Hager^9^ | 2006 | n=80 | 10 (±1.0), 50% male | USA | 5.30-6pm | Cross-sectional | Not reported | Parent report TV and computer/ video game use | Low Quality / High Bias |
| Harding et al.^49^ | 2015 | n= 363 | 12.0 (±0.4), 39% male | UK | End of school bell - 9pm | Cohort | Not reported | Actigraph accelerometer GT1M, SED<100cpm | Low Quality / High Bias |
| Harrison et al.^46^ | 2015 | n=283 | 9-10 years, 44.9% male | UK | 4-9pm | Cohort | Not reported | Actigraph accelerometer GT1M, 5 sec epoch SED<100cpm | Low Quality / High Bias |
| Hswen et al.^44^ | 2014 | n=477 | 12.7 (±0.91), 50% male | USA | NR | Cross-sectional data from intervention | Not reported | Self-report TV, video game and computer/internet | Low Quality / High Bias |
| Jago et al.^40^ | 2005 | n= 81 | 13.3 (±0.5), 54% male | USA | 3pm-6.59pm | Cross-sectional | Not reported | Actigraph MTI SED<800cpm, and self-report previous day electronic recreation recall | Mod Quality / Mod Bias |
| Jago et al.^45^ | 2008 | n=2670 | Year 3 and Year 9 students, 49% male | Denmark, Portugal, Estonia and Norway | Not reported | Cross-sectional | Not reported | Self-report TV | Low Quality / High Bias |
| Lau^23^ | 2013 | n=20 parent-child diads, | Children 6.25 (±0.64), 55% male | Australia | 3.30-7pm | Cross-sectional from cohort study | Not reported | Actical acceleromter, 15sec epoch,SED<100cpm | High Quality / Low Bias |
| Miller et al. ^41^ | 2012 | n=141 | 12.4 (±0.8), 28% male | USA | 3pm-12am | Cross-sectional | Variety | Self-report of sitting/lying activites | Low Quality / High Bias |
| Myers et al. ^46^ | 1996 | n=995 | 9-15 (45%male) | USA | Not reported | Cross-sectional | Not reported | Self-report (Self-Adminitered Physical Activity Checklist - SAPAC) | Mod Quality / Mod Bias |
| Newman et al.^24^ | 2007 | n=742 | 10.13, 48% male | Bulgaria, Taiwan and the USA | End of school until 10pm | Cross-sectional | Not reported | Self-report | Low Quality / High Bias |
| Orlowski et al.^25^ | 2010 | n=168 | 8.2 (±1.6), 50% male | USA | Not reported | Cross-sectional | After-school care | Self-report light/sitting activities | Low Quality / High Bias |
| Posner et al.^26^ | 1999 | n=194 (at 2-year follow-up) | 9.1 (±0.5), 54% boys | USA | 2.45-6pm or 3.35-6.35pm (dependant on dismissal time) | Cohort | Variety | Self-report | Mod Quality / Mod Bias |
| Pratt et al.^42^ | 1999 | n=1458 | 12 (±0.5), female sample | USA | 2pm onwards | Cross-sectional | Not reported | Actigraph 7165, 30sec epoch SED≤50cpm | Low Quality / High Bias |
| Pulsford et al.^27^ | 2013 | n= 629 | 10.95 (±0.4), 51% male | United Kingdom | 3-11pm | Cross-sectional | Not reported | Actigraph GT1M (Wrist worn), 10sec epoch, SED<100cpm | Low Quality / High Bias |
| Ramirez-Rico et al.^37^ | 2014 | n = 476 | 10-14, 43% male | England and Spain | 3hrs immediately after school | cross-sectional | Not reported | Actigraph GT1M, 5sec epoch, SED<100cpm | Low Quality / High Bias |
| Rosenkranz et al.^28^ | 2011 | n=240 | 9.3 (±0.7), 51% male | USA | Not reported | Cross-sectional | After-school care | Actigraph GT1M, 30sec epoch, SED<100cpm | Low Quality / High Bias |
| Silva et al.^43^ | 2011 | n=24 | 11.04 (±1.45), 50% males | Portugal | 6.01pm-8pm | Cross-sectional | Not reported | Actigraph, 7164, 60sec epoch, SED<50cpm | Mod Quality / Mod Bias |
| Stone et al.^36^ | 2014 | n=856 | 11 (±0.6), 45% males | Canada | 2hrs after school bell | Cohort | Not reported | Actigraph GT1M, 5sec epoch, SED<300cpm | Low Quality / High Bias |
| Stone and Faulkner^35^ | 2014 | n=856 | 11 (±0.6), 45% males | Canada | 2hrs after school bell | Cohort | Not reported | Actigraph GT1M, 5sec epoch, SED<300cpm | Low Quality / High Bias |
| Taverno Ross et al.^29^ | 2012 | n=662 | 10.6 (±0.5), 45% male | USA | after school bell - 6pm | Cross-sectional | Variety | Actigraph GT1M and GT3Xm, 60sec epoch, SED<100cpm | Mod Quality / Mod Bias |
| Trost et al.^30^ | 2008 | n=147 | 10.1 (±0.7), 54% male | USA | Not reported | Cross-sectional | After-school care | Actigraph GT1M, 30sec epoch, SED<1.5METs | Mod Quality / Mod Bias |
| Vissers et al.^31^ | 2011 | n=1697 | 10.3 (±0.3), 44% males | England | 12noon - 9pm | Cross-sectional | Not reported | Actigraph GT1M, 5sec epoch, SED<100cpm | Low Quality / High Bias |
| Wen et al.^32^ | 2009 | n=1974 | grade 5-6, 47% male | Australia | Not reported | Cross-sectional | Not reported | Self-report screen time recall | Low Quality / High Bias |
| Wickel.^33^ | 2013 | n=862 year 3 students and 954 year 4 students | 10, 50% male | USA | 3-6pm | Cohort | Variety | Self-report present day screen- and non-screen based sedentary behavior recall | Mod Quality / Mod Bias |
| Wickel et al.^34^ | 2013 | n=886 | 9-11, 50% male | USA | 3-6pm | Cohort | Variety | Self-report present day screen- and non-screen based sedentary behavior recall | Mod Quality / Mod Bias |
